# Retrospective real-world analysis of adherence and persistence to lipid-lowering therapy in Germany

**DOI:** 10.1007/s00392-023-02257-6

**Published:** 2023-08-21

**Authors:** Wolfgang Koenig, Elke S. Lorenz, Lea Beier, Ioanna Gouni-Berthold

**Affiliations:** 1 Deutsches Herzzentrum München, Technische Universität München, Munich, Germany; 2 DZHK (German Centre for Cardiovascular Research), Partner Site Munich Heart Alliance, Munich, Germany; 3 Institute of Epidemiology and Medical Biometry, University of Ulm, Ulm, Germany; 4 Novartis Pharma GmbH, Nuremberg, Germany; 5 Center for Endocrinology, Diabetes, and Preventive Medicine, Faculty of Medicine, University of Cologne, University Hospital Cologne, Cologne, Germany

**Keywords:** Persistence, Adherence, Germany, Lipid-lowering therapy, Statins, Ezetimibe, Anti-PCSK9 antibody

## Abstract

**Background:**

Cardiovascular disease is the leading cause of mortality in Germany. Cardiovascular risk can be mitigated with long-term lipid-lowering therapies (LLTs) that reduce levels of low-density lipoprotein cholesterol. Although effective, risk mitigation is hindered by poor persistence and adherence.

**Objective:**

To investigate real-world persistence and adherence to LLTs through 36 months post-initiation.

**Methods:**

This retrospective cohort study included patients with dyslipidemia who were newly prescribed LLTs between July and December 2017, using anonymized prescription data from the Insight Health™ Patient Insight Tool, and followed up until March 2021. Persistence and adherence to the therapies were stratified by age and sex. The proportion of days covered (PDC) was used to measure adherence.

**Results:**

Patients with dyslipidemia and newly prescribed statins (*n* = 865,732), ezetimibe (*n* = 34,490), or anti-proprotein convertase subtilisin/kexin type 9 monoclonal antibodies (anti-PCSK9 mAbs; *n* = 1940) were included. Persistence to LLTs declined gradually across all treatment subgroups and was lower in women than men. Adherence, calculated as the mean PDC at the end of the analysis period (July 2017‒March 2021) was 0.84, 0.92, and 0.93 for statins, ezetimibe, and anti-PCSK9 mAbs, respectively. Among patients who discontinued treatment, mean treatment duration was 265, 255, and 387 days for statins, ezetimibe, and anti-PCSK9 mAbs, respectively. Only ~ 10% of patients persisted between 201 and 300 days. By Day 300, 71% of patients on statins had discontinued treatment. At 36 months, overall persistence rates were lowest with statins (20.6%), followed by ezetimibe (22.3%) and anti-PCSK9 mAbs (50.9%).

**Conclusions:**

High non-persistence rates were observed across all LLT regimens analyzed, with the lowest persistence rates observed with statins.

**Graphical Abstract:**

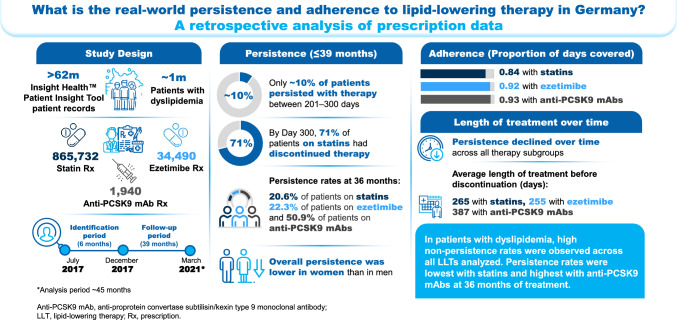

**Supplementary Information:**

The online version contains supplementary material available at 10.1007/s00392-023-02257-6.

## Introduction

In Germany, an estimated 34% of total deaths are caused by cardiovascular (CV) disease, and ischemic heart disease remains the leading cause of mortality [1]. Elevated low-density lipoprotein cholesterol (LDL-C) represents a causal risk factor for the development of atherosclerotic cardiovascular disease (ASCVD) [2]. A reduction in LDL-C levels by lipid-lowering therapies (LLTs) is consistently associated with a lower risk of CV events such as myocardial infarction, peripheral arterial disease, and ischemic stroke [3–5]. Treatment for dyslipidemia with LLTs is a long-term, usually lifelong, and risk-adapted approach that involves regular monitoring to achieve and maintain target LDL-C levels [2]. The 2019 European Society of Cardiology/European Atherosclerosis Society guidelines for the management of dyslipidemia and the 2018 American Heart Association guidelines on the management of blood cholesterol suggest assessing total CV risk and baseline LDL-C levels to define the treatment goal [2, 6]. Together with lifestyle modifications, maximum tolerated doses of high-intensity statins are recommended to reach target LDL-C goals. Additional LLTs such as ezetimibe and proprotein convertase subtilisin/kexin type 9 monoclonal antibodies (anti-PCSK9 mAbs) are needed to treat patients whose LDL-C goals are not achieved despite the use of the maximum tolerated statin therapy [2].

Adherence and persistence to pharmacotherapies have been shown to be key drivers for achieving therapeutic goals and improving clinical outcomes [7]. Multiple factors contribute to LLT nonadherence, such as healthcare disparities due to socioeconomic status, age, race, and sex; cost and limited access to healthcare; perceived side effects associated with LLT; health literacy; and the presence of comorbidities [8]. Several real-world studies have shown that most patients do not achieve LDL-C goals due to low adherence and persistence to their prescribed LLTs [9–12]. However, data on long-term patient adherence and persistence to LLTs in Germany are scarce. To our knowledge, this is the first study to investigate real-world adherence and persistence to LLTs in patients newly treated with any lipid-lowering agent in Germany, by means of analyzing prescription data.

## Methods

### Data source

The study used anonymized, double-encrypted prescription data from the Insight Health™ Patient Insight Tool, covering over 62 million outpatients with an electronic health card as of January 2017, which covered approximately 77% of the outpatient statutory health insurance prescription market in Germany. Redeemed prescriptions were submitted via pharmacy data processing centers. In this analysis, data from patients who were newly prescribed LLTs between July and December 2017 were collected from all 16 federal states. The analysis period was from July 2017 to March 2021. As this analysis represents the secondary use of databases with anonymized data, ethics committee approval was not needed.

### Study design and objectives

Patients who were newly prescribed at least one LLT, and who were followed up for 39 months until March 2021, were included. First-time prescriptions included 865,732 statin prescriptions, stratified by dosing (low, medium, or high doses of atorvastatin, fluvastatin, lovastatin, pitavastatin, pravastatin, rosuvastatin, and simvastatin; Supplementary Table 1), 34,490 ezetimibe prescriptions and 1940 anti-PCSK9 mAbs (alirocumab, evolocumab) prescriptions, or a fixed-dose combination of statin plus ezetimibe (simvastatin/atorvastatin/rosuvastatin + ezetimibe) (Supplementary Fig. 1).

With respect to concomitant medication at baseline, 98% of patients newly prescribed statins were on at least one, as were 99% of patients newly prescribed ezetimibe or anti-PCSK9 mAbs.

The main objective of this study was to describe the adherence and persistence of patients to these LLT regimens.

Patients who discontinued treatment were followed up to record any subsequent treatment received after discontinuation of LLT. To guarantee a follow-up of 6 months, only patients who had received their last prescription by September 2020 were considered.

### Data analysis

Persistence was based on the proportion of patients remaining on their prescribed LLT beyond their first prescription for a prespecified amount of time. Persistence rates for each LLT regimen were further analyzed by sex, age group (< 50 years, 50–69 years,  ≥ 70 years), statin intensity and number of concomitant medications at baseline. Non-persistence was defined as LLT discontinuation with a ≥ 90-day prescription gap between the end of the initial prescription and the dispensation of a new one. A sensitivity analysis for persistence was conducted using a prescription gap of 180 days, in line with previously published studies [13, 14]. In such cases, treatment duration was analyzed for each of the LLT regimens. Patients who were non-persistent were not included in the calculations.

Adherence to an LLT was measured as the proportion of days covered (PDC) in patients who had filled ≥ 2 successive prescriptions. This assessment did not consider any excess medication packs, i.e., that the patient collected the prescription but did not use it all. The PDC was thus based on the assumption that the patient had either used up their previous medication pack or the new prescription had replaced it, such that the maximum number of packs that each patient had at their disposal at any given time was one (PDC ≤ 1). The PDC was calculated for each patient by examining their last identifiable prescription until the end of the analysis period in March 2021.

Descriptive summary statistics were derived for patient adherence and persistence. All data analyses were conducted by Insight Health™ GmbH & Co KG.

## Results

### Patient selection

This retrospective study included patients who had been newly prescribed statins (*n* = 865,732), ezetimibe (*n* = 34,490), or anti-PCSK9 mAbs (*n* = 1940), or a combination of these between July and December 2017. The analyses reported here were carried out on this patient cohort. Patients were followed up between July 2017 and March 2021.

### Patient characteristics

Patient characteristics, including their initial prescription, are shown in Table 1. In summary, 82% of patients were ≥ 60 years of age. The proportion of men and women was comparable across the statin, ezetimibe, and anti-PCSK9 mAb treatment subgroups. At baseline, approximately 98% of patients on statins (*n*/*N* = 852,710/865,732) and 99% of patients on ezetimibe (*n*/*N* = 34,204/34,491) and anti-PCSK9 mAbs (*n*/*N* = 1915/1940) were on concomitant medication (ranging from 1 to ≥ 21 drugs) at the time of analysis; 28%, 27%, and 22% of patients on statins, ezetimibe, and anti-PCSK9 mAbs, respectively, were on antidiabetic medication. At baseline, over 60% of the patients were receiving 7–15 different concomitant medications along with LLTs.

**Table 1:** Patient characteristics

Parameter	Statin (*n* = 865,732)	Ezetimibe (*n* = 34,490)	Anti-PCSK9 mAb (*n* = 1940)
Age range (years), %			
< 50	4.1	3.9	6.4
50–69	38.2	45.1	56.1
≥ 70	57.5	50.9	37.5
Unknown	0.2	0.1	0.0
Sex, %			
Male	44.3	50.3	49.2
Female	42.9	38.4	40.6
Unknown	12.8	11.2	10.2
Patients on any comedication (%)^a^	*n* = 852,710 (98.5)	*n* = 34,204 (99.2)	*n* = 1915 (98.7)
Comedication, %			
ACEi	39.5	39.0	31.9
ARB	25.1	29.4	39.6
Cholesterol and triglyceride-lowering medication^b^	–	77.2	72.8
Beta-blocker	54.4	64.6	68.9
Antithrombotic agent	34.3	46.8	59.4
Diuretic	36.7	36.4	37.0
Calcium channel blocker	32.4	30.2	31.9
Antidiabetic	27.7	27.2	22.0
Anti-ulcer agents^c^	49.3	51.6	59.3
Anti-inflammatory and antirheumatic agents^d^	44.7	39.5	46.6
Number of comedications, %			
0	1.5	0.8	1.3
1–3	14.1	9.8	5.5
4–6	20.5	19.4	13.4
7–10	26.4	28.0	24.0
11–15	22.0	23.8	28.0
16–20	10.2	11.2	16.6
≥ 21	5.4	6.9	11.2

*ACEi* angiotensin-converting enzyme inhibitor; *ARB* angiotensin receptor blocker; *anti-PCSK9 mAb* proprotein convertase subtilisin/kexin type 9 monoclonal antibody

^a^Data on co-medication is not available for all patients

^b^Alirocumab, atorvastatin, bempedoic acid, bempedoic acid + ezetimibe, bezafibrate, colesevelam, colestyramine, colestyramine 20 (12% water), evolocumab, ezetimibe, fenofibrate, fluvastatin, gemfibrozil, inclisiran, lovastatin, pitavastatin, pravastatin, rosuvastatin, simvastatin, volanesorsen

^c^Calcium carbonate + magnesium carbonate + opuntia ficus-indica (L.) Mill., cimetidine, citric acid, bismuth potassium salt (2:1:5) – 1.5 water + tetracycline + metronidazole, dexlansoprazole, esomeprazole, famotidine, limestone, ground (7W), lansoprazole, misoprostol, omeprazole, omeprazole + amoxicillin + clarithromycin; pantoprazole; pantoprazole + amoxicillin + clarithromycin; pirenzepine; rabeprazole; ranitidine; sucralfate

^d^Non-steroids

### Treatment persistence

**Fig. 1:**
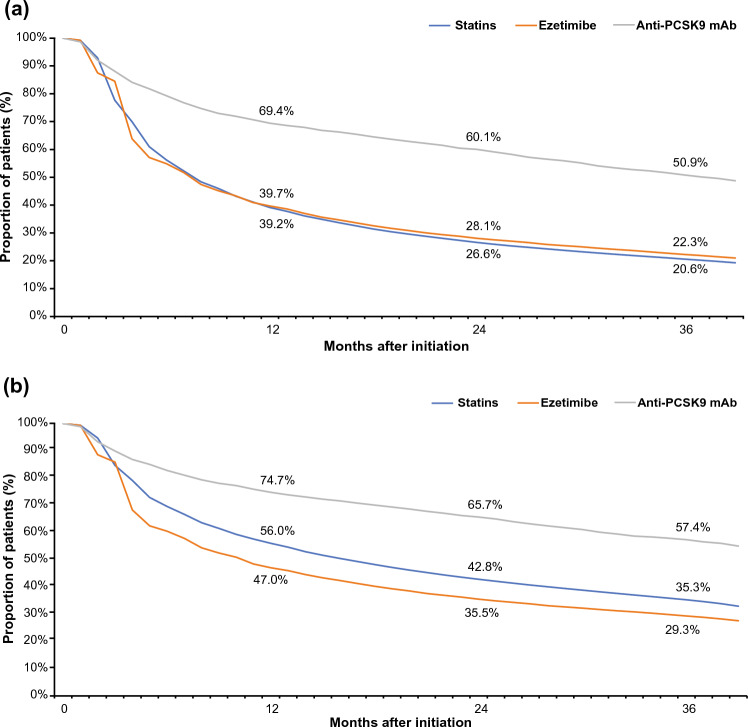
Treatment persistence over time. **a** Treatment persistence for up to 39 months of follow-up using the 90-day discontinuation criterion. **b** Sensitivity analysis of treatment persistence for up to 39 months of follow-up using the 180-day discontinuation criterion. *Anti-PCSK9 mAb* anti-proprotein convertase subtilisin/kexin type 9 monoclonal antibody

Persistence to any LLT regimen included in this analysis (statin, ezetimibe, and anti-PCSK9 mAb) declined gradually across all three treatment subgroups during the study period. Thirty-six months after the initial prescription, only 20.6% of patients on statins, 22.3% of those on ezetimibe, and 50.9% of those on anti-PCSK9 mAbs remained on treatment (Fig. 1a).

Sensitivity analysis with a 180-day prescription gap criterion showed a consistent trend with a time-dependent decline in LLT persistence across all cohorts (Fig. 1b).

Among patients who were prescribed a statin, those on low- (*n* = 76,836) or moderate-intensity (*n* = 716,315) treatment were more likely to discontinue treatment at the end of 36 months than patients on high-intensity (*n* = 185,829) treatment. Patients who initiated low-intensity statins had the lowest rates of persistence (4.0%; Supplementary Fig. 2) and the highest rates of treatment discontinuation (85.5%). Conversely, patients who initiated treatment with high-intensity statins had the highest persistence (41.5%; Supplementary Fig. 2) and, subsequently, the lowest treatment discontinuation rate (54.9%). Persistence and treatment discontinuation rates in patients initiated on moderate-intensity statins were 15.3% and 82.8%, respectively. A total of 15.8% of patients were up-titrated to a higher dose (Supplementary Fig. 3). The proportion of patients whose statin dose was up-titrated was highest in the low- to moderate-intensity statin subgroup. Only 8.1% of patients receiving statins were down-titrated to a lower dose; this was mostly seen with patients on high-intensity statins switching to a moderate dose.

Persistence to any LLT was similar between diabetic patients on concomitant antidiabetic therapy and non-diabetics (statins, 20.9% vs 16.9%; ezetimibe, 22.7% vs 18.1%; and anti-PCSK9 mAb, 49.4% vs 45.4%, respectively), using the 90-day exclusion criterion. Persistence to any LLT increased with an increasing number of comedications (Supplementary Fig. 4). Patients who were receiving 0–3 comedications had the lowest rates of persistence to LLT (statins, 5.8%; ezetimibe, 3.5%; anti-PCSK9 mAb, 10.6%) and those receiving ≥ 7 comedications had the highest (statins, 26.5%; ezetimibe, 28.6%; anti-PCSK9 mAb, 57.8%) (Supplementary Fig. 4).

**Fig. 2:**
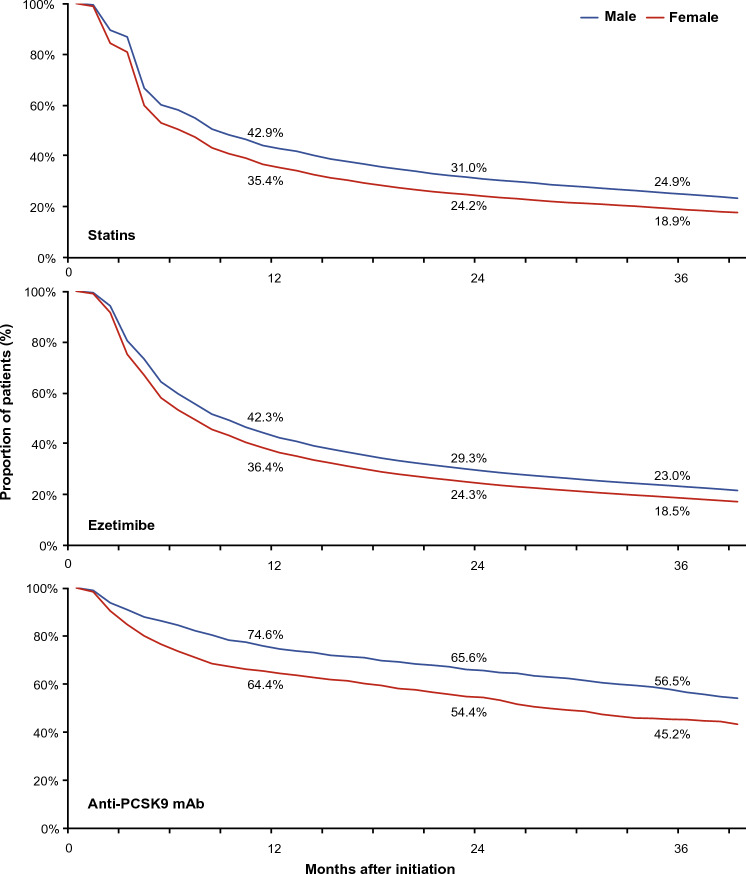
Treatment persistence over time stratified by sex^a^. ^a^Patients who did not disclose their sex at birth (sex ‘unknown’) were excluded from this analysis. *Anti-PCSK9 mAb* anti-proprotein convertase subtilisin/kexin type 9 monoclonal antibody

**Fig. 3:**
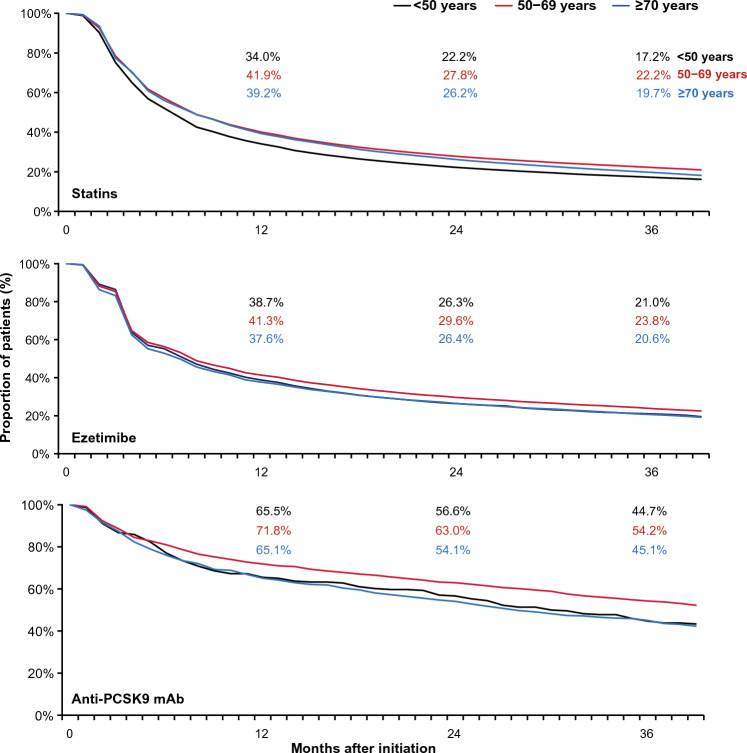
Treatment persistence stratified by age^a^. ^a^Analysis was done using the 90-day discontinuation criterion. *Anti-PCSK9 mAb* anti-proprotein convertase subtilisin/kexin type 9 monoclonal antibody

Overall, persistence rates to LLTs were lower in women than in men across all regimens (Fig. 2), with persistence to statins and ezetimibe being approximately 5% lower for women, and 10% lower for anti-PCSK9 mAbs, compared with men. However, over 36 months, and at all time points, persistence rates to statins and ezetimibe were similar across age groups, except for anti-PCKS9 mAbs, where persistence was higher in the 50‒69 years age group compared with the < 50 years, or ≥ 70 years age groups (Fig. 3).

### Treatment adherence

Adherence, calculated as the proportion of days out of 100 that patients adhered to their treatment regimen, was 92/100 days for patients on ezetimibe, 93/100 for patients on anti-PCSK9 mAbs, and was lower for patients who were prescribed statins (84/100 days); adherence was generally high until treatment discontinuation within the individual therapy period (Supplementary Table 2).

**Fig. 4:**
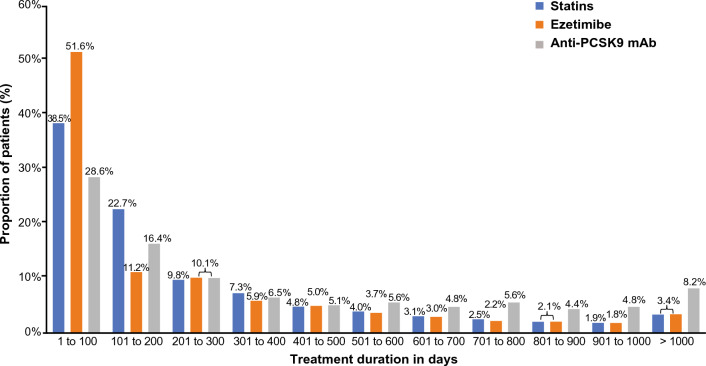
Treatment duration in patients who discontinued treatment^a^. ^a^Analysis was done using the 90-day discontinuation criterion. The analysis period was from July 2017 to March 2021 (approx. 45 months) and considers that the duration of therapy may have been extended (e.g., if multiple prescriptions were issued) if the patient continued therapy beyond the prescribed time period. *Anti-PCSK9 mAb* anti-proprotein convertase subtilisin/kexin type 9 monoclonal antibody

Of those patients who did not persist with their prescribed therapy in the long-term, approximately 10% had a therapy duration of 201–300 days (Fig. 4). In these patients, the average treatment duration was 265 days (± SD, 273) in the statin subgroup, 255 days (± SD, 273) in the ezetimibe subgroup, and 387 days (± SD, 353) in the anti-PCSK9 mAb subgroup. Overall, 71%, 72.9%, and 55% of patients on statins, ezetimibe, and anti-PCSK9 mAbs, respectively, discontinued their prescribed LLT by the 300-day time point. Patients who were prescribed another LLT following discontinuation are shown in Supplementary Table 3.

## Discussion

Clinical evidence and treatment guidelines recommend LLTs to reduce elevated LDL-C levels and the associated risk of ASCVD; however, poor adherence and persistence to LLT regimens remain a significant concern [2, 9]. In this retrospective longitudinal study, we report an in-depth analysis of real-world adherence and persistence to LLTs in Germany by analyzing prescription data from patients who were newly prescribed statins, ezetimibe, or anti-PCSK9 mAbs by their physician between July and December 2017, and who were followed up until March 2021. We observed low rates of persistence and high treatment discontinuation rates across all LLT regimens.

A retrospective analysis of the Optum Research Database in the United States reported that statin persistence in primary (general and high-risk populations) and secondary prevention populations ranged from 7% to 84% over a follow-up period of 3–36 months [11]. These findings were in accordance with those from the present study, in which persistence to an LLT regimen declined gradually during the study period and, at 36 months, only ~ 21% of patients remained on statin treatment, ~ 22% on ezetimibe, and ~ 51% on anti-PCSK9 mAbs. Analyses of practice data from ~ 2.6 million patient records from the IMS Disease Analyzer database in Germany in 2017 indicated that < 20% of patients received high-intensity statins or statin therapy in any dose in combination with other LLTs [15]. Of the patients receiving LLTs, > 80% who were at high risk of CV disease did not achieve their target LDL-C levels. Many factors may cause this, such as suboptimal adherence and persistence.

Similar studies have indicated low long-term persistence rates among patients prescribed statins and ezetimibe [11, 16]. In this study, we found that patients who initiated treatment with high-intensity statins had the highest persistence rates. Use of high-intensity statins is normally indicated for the treatment of high LDL-C levels or when lower LDL-C goals in high-risk patients are required. Compared to a primary prevention population, patients with established ASCVD are more likely to receive statin therapy, particularly high-intensity statins [17]. In our study, patients who received high-intensity statins and who were, presumably, more likely to be patients with either established ASCVD or at high risk of ASCVD, showed the highest persistence rates. Interestingly, the persistence rate increased with an increase in the number of comedications, which indicates that patients with severe disease and/or multiple comorbidities who are receiving many comedications are more persistent to LLTs. This could be probably due to frequent follow-ups with the physician and being mindful of their high CV risk.

Our results were in agreement with recent findings from a 2010–2016 retrospective observational cohort study in Sweden [18]. The generally low persistence rates with statins observed in our study were concerning, as discontinuation of statin therapy can increase the risk of CV events considerably [19]. An Italian study with patients newly prescribed anti-PCSK9 mAbs during their first year of availability reported that 73% of patients were continuing with the therapy 6 months after treatment initiation; however, long-term real-world evidence data were lacking in this study [20].

Poorer adherence and persistence to LLTs such as statins have been associated with women [11, 21]. Results from our study were in line with published findings: women who were prescribed statins and ezetimibe had an approximately 5% lower persistence rate compared with men at all time points, and this difference was ~ 10% for those prescribed anti-PCSK9 mAbs. Lower prescription rates and awareness of CV risks among women may have contributed to lower persistence rates than among men [22–24]. Previous studies from the United States showed that compared with men, women are more likely to discontinue or switch statins due to statin-related (or perceived) side effects [25, 26].

Early screening and LLT initiation in eligible patients can lead to significant clinical benefits in the long term. However, there is still a tendency to prescribe LLTs at later stages of life, as seen in this analysis; a high proportion (~ 82%) of patients were 60 years of age or older at baseline. We observed that age had a U-shaped association with persistence to statins, ezetimibe, and anti-PCSK9 mAbs; patients in the 50–69 years age group had the highest persistence rates compared with those aged < 50 years or ≥ 70 years at all time points.

To our knowledge, our study represents the first real-world analysis of adherence and persistence data in German patients with dyslipidemia who were newly prescribed statins, ezetimibe, or an anti-PCSK9 mAb. Our data demonstrate low persistence rates to long-term therapy irrespective of lipid-lowering agent, and independently from any insurance coverage or reimbursement plans.

Adherence to pharmacotherapies can help achieve guideline-recommended LDL-C goals and thereby reduce long-term CV outcomes in high-risk patients; however, poor adherence limits the benefit of such therapies [19]. A recent meta-analysis of real-world lipid management studies across Europe showed that adherence to LLTs was variable and ranged between 46% and 92% [27], which was lower than our data suggest, with adherence ranging between 84% and 93%.

It is a cause for concern that despite the proven benefits of guideline-recommended therapies, therapy duration was between 201–300 days (less than one year) for approximately 10% of patients who discontinued their prescribed therapy, in contrast to the expectation of lifelong therapy to minimize the risk of CV events. Considering that long-term persistence to anti-PCSK9 mAbs was approximately 50% in this analysis, which is ~ 2.5 times higher than that for statins and ezetimibe, and that they are administered monthly or twice-monthly, as opposed to daily, therapeutics such as inclisiran, which is administered even less frequently, could help with increasing patient persistence in the long-term—especially in populations with elevated LDL-C levels at high risk of CV events [28]. Implementation of initiatives and digital tools for educating both patients and physicians, pharmacy-based programs designed to help patients with prescription refills, including reminders, nurse-based long-term follow-up with dosing titrations by telephone, and the active involvement of patients in tracking LDL-C target levels (using patient cards) have been shown to be effective in improving LLT adherence [8, 29, 30].

The current study analyzed retrospective data from a single database in Germany. A wider analysis based on a similar approach from multiple databases in different federal states would help to provide a better overview of patients’ approaches to LLTs. Further investigation is warranted to recognize factors that may improve medication adherence and persistence.

### Limitations

This study has several limitations. Prescription dispensation was used as a proxy for medication use by the patient. Data are focused on outpatients and represent a potential selection bias due to the required multi-year follow-up. Clinical parameters, as well as other sensitive medical information such as a patient’s diagnosis or comorbidities were not provided on prescription slips and, therefore, not collected by pharmacy coding centers. As such, this information could not be presented as part of this analysis. Although data on subsequent therapy were captured, the reasons for switching were not available. Lipid-lowering therapy segments were looked at separately, such that patients’ entries were counted twice if they had received two LLTs within the 6-month period (July–December 2017). The PDC may only show primary adherence, how often medications are filled by the automated system and picked up by patients rather than the intended capture of proper and consistent medication usage. Pharmacies and insurance plans can alter refill requirements, thus artificially affecting PDC by changing the quantity allowed per prescription and authorized fill dates.

## Conclusions

High rates of non-persistence were observed across all LLT subgroups, with a high proportion of patients discontinuing their prescribed LLTs within 300 days of therapy initiation. Anti-PCSK9 mAbs had the highest persistence rates over 36 months (with ~ 50% of patients persisting), compared with statins and ezetimibe. Overall, adherence to LLT regimens was higher among patients who were prescribed ezetimibe and anti-PCSK9 mAbs than among those who were prescribed statins. This highlights the need for improved adherence and persistence to LLTs in Germany. Further studies are needed to understand the drivers of non-persistence in Germany.

### Supplementary Information

Below is the link to the electronic supplementary material.

Supplementary file1 (DOCX 274 KB)

## Data Availability

Raw data are not openly available due to data privacy laws that govern its dissemination, and due to data ownership considerations. Analyzed data that support the findings of this study can be made available upon request.

## References

[1] German Federal Statistical Office: causes of death statistics. https://www.destatis.de/EN/Press/2021/11/PE21_505_23211.html (2020). Accessed 19 June 2023

[2] Mach F, Baigent C, Catapano AL, Koskinas KC, Casula M, Badimon L (2020). 2019 ESC/EAS guidelines for the management of dyslipidaemias: lipid modification to reduce cardiovascular risk. Eur Heart J.

[3] Cholesterol Treatment Trialists’ (CTT) Collaboration (2010) Efficacy and safety of more intensive lowering of LDL cholesterol: a meta-analysis of data from 170,000 participants in 26 randomised trials. Lancet 376(9753):1670–1681. 10.1016/S0140-6736(10)61350-5 10.1016/S0140-6736(10)61350-5 PMC2988224 21067804

[4] Ference BA, Ginsberg HN, Graham I, Ray KK, Packard CJ, Bruckert E et al (2017) Low-density lipoproteins cause atherosclerotic cardiovascular disease. 1. Evidence from genetic, epidemiologic, and clinical studies. A consensus statement from the European Atherosclerosis Society Consensus Panel. Eur Heart J 38(32):2459–2472. 10.1093/eurheartj/ehx144 10.1093/eurheartj/ehx144 PMC5837225 28444290

[5] Ahrens I, Khachatryan A, Monga B, Dornstauder E, Sidelnikov E (2021). Association of treatment intensity and adherence to lipid-lowering therapy with major adverse cardiovascular events among post-MI Patients in Germany. Adv Ther.

[6] Grundy SM, Stone NJ, Bailey AL, Beam C, Birtcher KK, Blumenthal RS (2019). 2018 AHA/ACC/AACVPR/AAPA/ABC/ACPM/ADA/AGS/APhA/ASPC/NLA/PCNA guideline on the management of blood cholesterol: a report of the American College of Cardiology/American Heart Association Task Force on Clinical Practice Guidelines. J Am Coll Cardiol.

[7] Cramer JA, Benedict A, Muszbek N, Keskinaslan A, Khan ZM (2008). The significance of compliance and persistence in the treatment of diabetes, hypertension and dyslipidaemia: a review. Int J Clin Pract.

[8] Desai NR, Farbaniec M, Karalis DG (2023). Nonadherence to lipid-lowering therapy and strategies to improve adherence in patients with atherosclerotic cardiovascular disease. Clin Cardiol.

[9] Klimis H, Chow CK (2019). Clinical consequences of poor adherence to lipid-lowering therapy in patients with cardiovascular disease: can we do better?. Heart Asia.

[10] Ofori-Asenso R, Jakhu A, Zomer E, Curtis AJ, Korhonen MJ, Nelson M (2018). Adherence and persistence among statin users aged 65 years and over: a systematic review and meta-analysis. J Gerontol A Biol Sci Med Sci.

[11] Toth PP, Granowitz C, Hull M, Anderson A, Philip S (2019). Long-term statin persistence is poor among high-risk patients with dyslipidemia: a real-world administrative claims analysis. Lipids Health Dis.

[12] Wake M, Oh A, Onishi Y, Guelfucci F, Shimasaki Y, Teramoto T (2019). Adherence and persistence to hyperlipidemia medications in patients with atherosclerotic cardiovascular disease and those with diabetes mellitus based on administrative claims data in Japan. Atherosclerosis.

[13] Hardtstock F, Maywald U, Timmermann H, Unmussig V, Muller S, Wilke T (2022). Extent of non-adherence and non-persistence in asthma patients: analysis of a large claims data set. J Asthma.

[14] Wilke T, Groth A, Fuchs A, Pfannkuche M, Maywald U (2017). Persistence with VKA treatment in newly treated atrial fibrillation patients: an analysis based on a large sample of 38,076 German patients. Eur J Clin Pharmacol.

[15] Kostev K, Parhofer KG, Dippel FW (2017). Prevalence of high-risk cardiovascular patients with therapy-resistant hypercholesterolemia. Cardiovasc Endocrinol.

[16] Santoleri F, Romagnoli A, Costantini A (2021). Adherence and persistence in the use of statins and ezetimibe over 8 years in a real-life study. Curr Med Res Opin.

[17] Chobufo MD, Regner SR, Zeb I, Lacoste JL, Virani SS, Balla S (2022). Burden and predictors of statin use in primary and secondary prevention of atherosclerotic vascular disease in the US: from the National Health and Nutrition Examination Survey 2017–2020. Eur J Prev Cardiol.

[18] Svensson MK, Sorio Vilela F, Leosdottir M, Banefelt J, Lindh M, Dun AR et al (2022) Effects of lipid-lowering treatment intensity and adherence on cardiovascular outcomes in patients with a recent myocardial infarction: a Swedish register-based study. Ups J Med Sci. 10.48101/ujms.v127.8296 10.48101/ujms.v127.8296 PMC9171571 35722183

[19] Bansilal S, Castellano JM, Garrido E, Wei HG, Freeman A, Spettell C (2016). Assessing the impact of medication adherence on long-term cardiovascular outcomes. J Am Coll Cardiol.

[20] Piccinni C, Antonazzo IC, Maggioni AP, Pedrini A, Calabria S, Ronconi G (2020). PCSK9 inhibitors' new users: analysis of prescription patterns and patients' characteristics from an Italian real-world study. Clin Drug Investig.

[21] Mann DM, Woodward M, Muntner P, Falzon L, Kronish I (2010). Predictors of nonadherence to statins: a systematic review and meta-analysis. Ann Pharmacother.

[22] Ballo P, Balzi D, Barchielli A, Turco L, Franconi F, Zuppiroli A (2016). Gender differences in statin prescription rates, adequacy of dosing, and association of statin therapy with outcome after heart failure hospitalization: a retrospective analysis in a community setting. Eur J Clin Pharmacol.

[23] Daponte-Codina A, Knox EC, Mateo-Rodriguez I, Seims A, Regitz-Zagrosek V, Maas A (2022). Gender and social inequalities in awareness of coronary artery disease in European countries. Int J Environ Res Public Health.

[24] Vogel B, Acevedo M, Appelman Y, Bairey Merz CN, Chieffo A, Figtree GA (2021). The Lancet women and cardiovascular disease commission: reducing the global burden by 2030. Lancet.

[25] Nanna MG, Wang TY, Xiang Q, Goldberg AC, Robinson JG, Roger VL (2019). Sex differences in the use of statins in community practice. Circ Cardiovasc Qual Outcomes.

[26] Karalis DG, Wild RA, Maki KC, Gaskins R, Jacobson TA, Sponseller CA (2016). Gender differences in side effects and attitudes regarding statin use in the understanding statin use in America and gaps in patient education (USAGE) study. J Clin Lipidol.

[27] Barrios V, Soronen J, Carter AM, Anastassopoulou A (2021). Lipid management across Europe in the real-world setting: a rapid evidence review. Curr Med Res Opin.

[28] Brandts J, Ray KK (2020). Low density lipoprotein cholesterol-lowering strategies and population health: time to move to a cumulative exposure model. Circulation.

[29] Huber D, Wiken C, Henriksson R, Soderstrom L, Mooe T (2019). Statin treatment after acute coronary syndrome: adherence and reasons for non-adherence in a randomized controlled intervention trial. Sci Rep.

[30] Makhmudova U, Samadifar B, Maloku A, Haxhikadrija P, Geiling JA, Romer R (2023). Intensive lipid-lowering therapy for early achievement of guideline-recommended LDL-cholesterol levels in patients with ST-elevation myocardial infarction (“Jena auf Ziel”). Clin Res Cardiol.

