# Supplementary material for: Retrospective real-world analysis of adherence and persistence to lipid-lowering therapy in Germany
Source: Clin Res Cardiol. 2023 Aug 21;113(6):812–21. doi: 10.1007/s00392-023-02257-6 (PMC11108924; doi:10.1007/s00392-023-02257-6)
Supplement: Supplementary file 1 — Supplementary file1 (DOCX 274 KB) [file 392_2023_2257_MOESM1_ESM.docx]

**Supplementary File**

**Retrospective real-world analysis of adherence and persistence to lipid-lowering therapy in Germany**

**Authors:** Wolfgang Koenig, MD^1,2*^; Elke S. Lorenz, MD^1^; Lea Beier, Dr.rer.medic.^3^; Ioanna Gouni-Berthold, MD^4^

**Author affiliations:** ^1^Deutsches Herzzentrum München, Technische Universität München, Munich, Germany, DZHK (German Centre for Cardiovascular Research), partner site Munich Heart Alliance, Munich, Germany; ^2^Institute of Epidemiology and Medical Biometry, University of Ulm, Ulm, Germany; ^3^Novartis Pharma GmbH, Nuremberg, Germany; **^4^**Center for Endocrinology, Diabetes, and Preventive Medicine, University of Cologne, Faculty of Medicine and University Hospital Cologne, Cologne, Germany

***Corresponding author:**Prof. Wolfgang Koenig

Deutsches Herzzentrum München, Technische Universität München, Munich, Germany, DZHK (German Centre for Cardiovascular Research), partner site Munich Heart Alliance, Munich, Germany, and Institute of Epidemiology and Medical Biometry, University of Ulm, Ulm, Germany

Phone: + 49-89-1218-4073

Email: [koenig@dhm.mhn.de](mailto:koenig@dhm.mhn.de); [wolfgang.koenig@uni-ulm.de](mailto:wolfgang.koenig@uni-ulm.de)

**Table of Contents**

[***Supplementary Fig. 1 Study design*** 3](#_Toc137809311)

[***Supplementary Fig. 2 Treatment persistence over time stratified by intensity of statin treatment^a^*** 3](#_Toc137809312)

[***Supplementary Fig. 3 Dose modification stratified by intensity of statin treatment*** 4](#_Toc137809313)

[***Supplementary Fig. 4 Treatment persistence over time stratified by number of comedications*** 5](#_Toc137809314)

[***Supplementary Table 1: Statin prescriptions stratified by dosing intensity*** 6](#_Toc137809315)

[***Supplementary Table 2. Adherence as a proportion of days covered*** 6](#_Toc137809316)

[***Supplementary Table 3. Subsequent therapy after discontinuation of LLT***^a^ 7](#_Toc137809317)

## ***Supplementary Fig. 1 Study design***


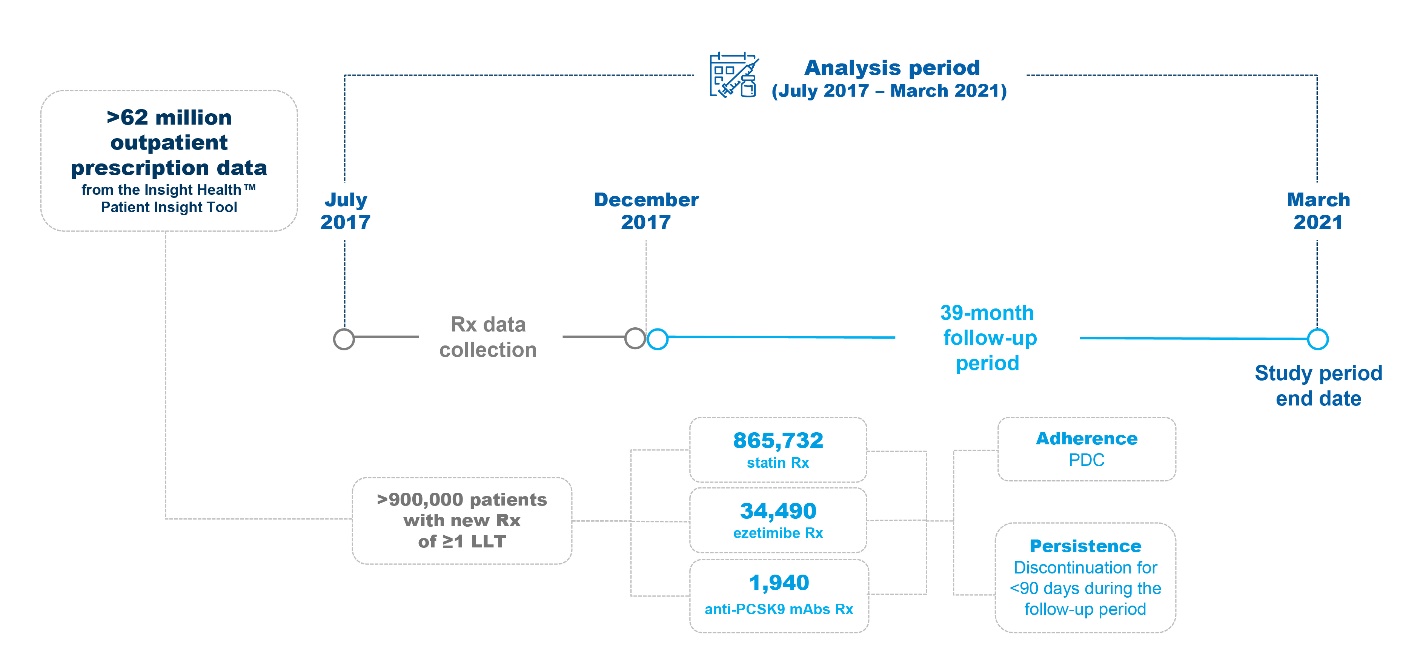


Anti-PCSK9 mAbs, proprotein convertase subtilisin/kexin type 9 monoclonal antibody; LLT, lipid-lowering therapy; PDC, proportion of days covered; Rx, prescription(s).

## ***Supplementary Fig. 2 Treatment persistence over time stratified by intensity of statin treatment^a^***

^a^Low-intensity statins included fluvastatin 20−40 mg, lovastatin 10−20 mg, pitavastatin 1−4 mg, simvastatin 5−10 mg, pravastatin 10−20 mg; Medium-intensity statins included atorvastatin 10−30 mg, fluvastatin 80 mg, lovastatin 40 mg, simvastatin 20−40 mg, pravastatin 30−40 mg, rosuvastatin 5−10 mg; High-intensity statins included atorvastatin 40−80 mg, simvastatin 60−80 mg, rosuvastatin 20−40 mg

## ***Supplementary Fig. 3 Dose modification stratified by intensity of statin treatment***

Patients were newly-prescribed low-intensity (n=76,836), moderate (n=716,315), or high-intensity statins (n=185,829).

## ***Supplementary Fig. 4 Treatment persistence over time stratified by number of comedications***

≥7

4–6

0–3

**Statins**

**Proportion of patients**

**Ezetimibe**


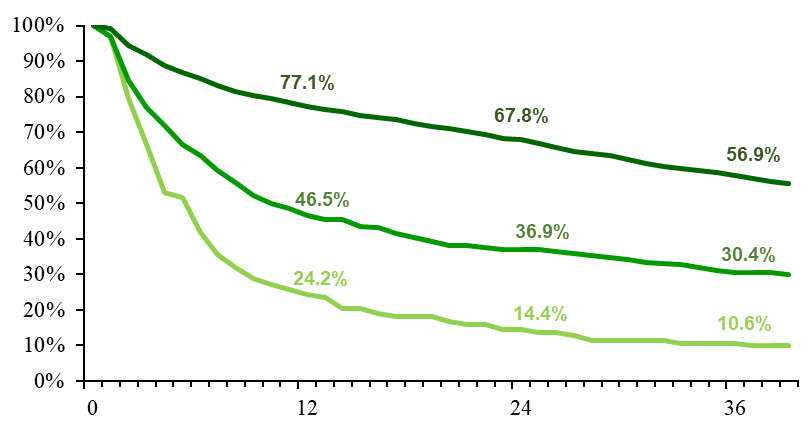


**Months after initiation**

**Anti-PCSK9 mAb**

Anti-PCSK9 mAb, anti-proprotein convertase subtilisin/kexin type 9 monoclonal antibody

## ***Supplementary Table 1: Statin prescriptions stratified by dosing intensity***

| **Statin** | **Low-intensity dose** | **Moderate-intensity dose** | **High-intensity dose** |
| --- | --- | --- | --- |
| Atorvastatin | - | 10−30 mg | 40−80 mg |
| Fluvastatin | 20−40 mg | 80 mg | - |
| Lovastatin | 10−20 mg | 40 mg | - |
| Pitavastatin | 1−4 mg | - | - |
| Simvastatin | 5−10 mg | 20−40 mg | 60−80 mg |
| Pravastatin | 10−20 mg | 30−40 mg | - |
| Rosuvastatin | - | 5−10 mg | 20−40 mg |

## ***Supplementary Table 2. Adherence as a proportion of days covered***

| **Treatment** | **Prescription Gap** | **Number** | **Mean (± SD)** |
| --- | --- | --- | --- |
| Statins | >90 days | 655,675 | 0.84 (0.16) |
| Ezetimibe | >90 days | 24,392 | 0.92 (0.09) |
| anti-PCSK9 mAb | >90 days | 1,757 | 0.93 (0.09) |

Abbreviations: Anti-PCSK9 mAb, anti-proprotein convertase subtilisin/kexin type 9 monoclonal antibody; SD, standard deviation.

Excess medication pack range (i.e., overlapping duration of prescription range) was not considered. The assumption was that either the patient had used up their previous pack or the new prescription had replaced the previous pack.

## ***Supplementary Table 3. Subsequent therapy after discontinuation of LLT***^a^

| **Type of LLT**  **prescribed** | **Number of dispensed prescriptions** | **Subsequent therapy after LLT discontinuation** | | |
| --- | --- | --- | --- | --- |
|  |  | **Statins No. (%)** | **Ezetimibe  No. (%)** | **Anti-PCSK9 mAb,**  **No. (%)** |
| Statins | 402,991 | - | 5,565 (1.4%) | 723 (0.2%) |
| Ezetimibe | 19,723 | 6,530 (33.1%) | - | 520 (2.6%) |
| Anti-PCSK9 mAb | 735 | 192 (26.1%) | 122 (16.6%) | - |

Abbreviations: Anti-PCSK9 mAb, anti-proprotein convertase subtilisin/kexin type 9-monoclonal antibody; LLT, lipid-lowering therapy.

^a^Each LLT prescription was treated as an individual segment and analyzed individually. In cases of simultaneous prescriptions of two LLTs, the patient appears in both columns, as the analysis is segment-naive

No further therapy: About 19%, 9%, and 14% of patients who discontinued the prescribed statin, ezetimibe, and anti-PCSK9 mAb treatment, respectively, did not receive any subsequent type of drug therapy, after discontinuation of prescribed LLT. Since no further prescriptions were documented for these patients, they were considered as lost to follow-up.
